# 

**Published:** 1915-04

**Authors:** 


### CONTENTS.

ORIGINAL COMMUNICATIONS—

The Treatment of Pyorrhea Alveolaris, With Special Reference to Emetine, by Robin Adair, M.D., D.D.S 1

Cardiac Hypertrophy and Dilatation, by M. T. Davis, M. D., Atlanta, Ga. .......... 12

The Epidemiology of Mumps, by Howard D. King, M. D., New Orleans, La. .......... 15

Meeting of the Fulton County Medical Society, by R. R. Daly, M. D. .......... 20

Atlanta Roentgen Ray Association, Regular Monthly Meeting, April 5, 1915 .......... 31

EDITORIAL .......... 35

SELECTIONS AND ABSTRACTS .......... 37

